# Comparative Analysis of Sex-Specific Aging

**DOI:** 10.1093/geroni/igaf122.1533

**Published:** 2025-12-31

**Authors:** Nicole Riddle

**Affiliations:** University of Alabama at Birmingham, Alabama, United States

## Abstract

Women and men show many differences in how they age, evident for example in the longer average lifespan seen in women. Sex-specific aging also occurs in many animal species. However, females do not always live longer. In some species males live longer than females, and in some species, there are no differences in lifespan between females and males despite other sex differences in aging. The Integration Initiative: Sex, Aging, Genomics, and Evolution (IISAGE) brings together researchers working in diverse species to exploit this naturally occurring variation in sex-specific patterns of aging to gain insights into the mechanistic basis of sex-specific aging and their evolution. The IISAGE team utilizes organismal, physiological, and genomics measures from various Drosophila species, moths and butterflies, fishes, geckos, turtles, snakes, bats, and mice. Using common methodologies allows us to carry out comparative studies across these diverse species. We present the results from several studies, including comparative transcriptomic analyses.

